# Stopping the Panel Doctor's Pay

**Published:** 1923-01

**Authors:** R. W. Harris

**Affiliations:** (formerly Assistant Secretary, responsible for the administration of National Health Insurance Medical Benefits, Ministry of Health)


### STOPPING THE PANEL DOCTOR'S PAY.

By R. W. HARRIS (formerly Assistant Secretary, responsible for the administration of National Health Insurance Medical Benefits, Ministry of Health).

IT is because I fear that the public are insufficiently informed of the steps taken to keep alive in the breast of the panel doctor what I will call the consciousness of his contract, that I have chosen the subject of this article. The most effective piece of machinery which the Minister of Health possesses for the control of the medical service for the insured is that which enables him to stop part of the doctor's pay if he has not earned it. That is putting it rather crudely. This is the position. Every doctor is paid, for treating his insured patients, out of a local fund which is itself a part of a central fund. This central fund contains (1) the amount available from the ordinary National Health Insurance funds, and (2) a supplementary amount which under special statutory authority is made the subject of conditions. These are simple, but comprehensive. The doctor must comply with his terms of service, and the standard of service must be satisfactory to the Minister. It will be seen that in a service financed out of public funds, public opinion would not be satisfied with less. If the Minister were to be guilty of arbitrary or unjust action he could always be called to account in the House of Commons, and doubtless the public press would not remain silent. As a matter of fact, so far as the press is concerned, the criticisms of the panel system which appear are certainly in the direction of calling for the more severe handling of the doctor who is in default, rather than greater leniency in the infliction of penalties.

The withholding of part of the doctor's pay has laid itself open to the objection, sometimes raised in interested quarters, that this is a fine, or a sort of infringement of the spirit of the Truck Acts. This is not the case. If you enter into a contract to carry out a certain piece of work, and, in doing so, to fulfil certain conditions, and you fail to observe those conditions, you thereby disentitle yourself to some part of the moneys provided for in the contract. And the panel doctor cannot have it both ways. If he accepted salaried service, after selection, he would come under the ordinary operation of the law and practice as between employer and employed. But if he remains more or less an independent contractor with the extraordinary right of being able to take part in this public service at his discretion and not at the discretion of the responsible authority, he must accept the conditions of his contract. I think that it is unquestionable that it is in the best interests of the service that with the Minister and not the local Insurance Committees should rest the responsibility for action in this matter of withholding remuneration, even if his constitutional position were not, as it is, unassailable. The standards of the different local bodies, in considering offences which have been committed, are so widely different that it is no exaggeration to say that for offences not apparently differing very appreciably in character or degree, one committee has reprimanded the doctor and ordered him to pay a trifling sum of expenses necessarily incurred by the insured person, and another has recommended the doctor's removal from the panel, which, if effected, would mean the loss of his livelihood, a loss which might be expressed, in terms of capital loss, in some thousands of pounds. It is to bridge the tremendous difference between these two penalties that the system obtains of withholding a lump sum from the doctor's pay. The amounts usually vary from £10 to £100, though sometimes larger or smaller amounts are involved.

I have known people to be greatly astonished that £50 has been withheld from a doctor's remuneration, who would have received unmoved the news that he had been removed from the panel. I have been told that a good doctor will not do his best work if he is in a constant state of apprehension that he is going to be hauled before a tribunal and, perhaps, fined £20 or £50 for what may be a genuine error of judgment. But I should be interested to learn of a single case in which this has happened. It is only in very rare cases, clear beyond any manner of question, that a penalty has been imposed for an error in diagnosis. And as regards the "tribunal," it is true that in 1921 I did hear one or two picturesque references to Star Chamber procedure and the like, but I feel sure that members of Panel Committees who have attended the hearings of doctors' "representations," which must be considered by the Minister before any penalty is imposed, will confirm my statement that there is not even the embarrassment of a Court about the procedure, still less of a Star Chamber. It is quite possible to be courteous and gentle with the doctor in question—shall we say, to observe towards him, in the circumstances, a "bedside manner"—even though you are afterwards proposing to stop £20 out of his pay.

Neglect of patients, carelessness in certification, the charging of fees, these are among the principal causes of default. I suppose a good doctor may most easily drop into carelessness in certification. But, advisedly, the whole system under which vast sums are paid out in sickness benefit, is built up on the foundation that every time a doctor gives a certificate he puts his signature to the statement, "I have examined you on the undermentioned date."

With regard to fee-charging there is a distinction between that niggling, microscopic examination of every service rendered, to see whether it comes within the insurance contract, which sometimes obtains and is bad enough, and the immoral acceptance of fees due to a distinction between panel and private service. It may be said that the patient insists on paying. The doctor should insist on refusing. He ought to make it clear that he can only render one kind of service, by whatever name it goes. If by so much as the raising of an eyebrow he lets it be understood that he could, if he would, give better treatment for a fee (I am not, of course, referring to specialist services), and that the panel service is poor stuff, he were better dead, figuratively speaking. I have more sympathy with a man who falls by way of the cup which cheers not wisely but too well.

